# Mitochondrial Dynamics in Drug-Induced Liver Injury

**DOI:** 10.3390/livers1030010

**Published:** 2021-06-23

**Authors:** Anup Ramachandran, David S. Umbaugh, Hartmut Jaeschke

**Affiliations:** Department of Pharmacology, Toxicology & Therapeutics, University of Kansas Medical Center, Kansas City, KS 66160, USA

**Keywords:** drug-induced liver injury, acetaminophen, mitochondrial dynamics, fusion, fission

## Abstract

Mitochondria have been studied for decades from the standpoint of metabolism and ATP generation. However, in recent years mitochondrial dynamics and its influence on bioenergetics and cellular homeostasis is also being appreciated. Mitochondria undergo regular cycles of fusion and fission regulated by various cues including cellular energy requirements and pathophysiological stimuli, and the network of critical proteins and membrane lipids involved in mitochondrial dynamics is being revealed. Hepatocytes are highly metabolic cells which have abundant mitochondria suggesting a biologically relevant role for mitochondrial dynamics in hepatocyte injury and recovery. Here we review information on molecular mediators of mitochondrial dynamics and their alteration in drug-induced liver injury. Based on current information, it is evident that changes in mitochondrial fusion and fission are hallmarks of liver pathophysiology ranging from acetaminophen-induced or cholestatic liver injury to chronic liver diseases. These alterations in mitochondrial dynamics influence multiple related mitochondrial responses such as mitophagy and mitochondrial biogenesis, which are important adaptive responses facilitating liver recovery in several contexts, including drug-induced liver injury. The current focus on characterization of molecular mechanisms of mitochondrial dynamics is of immense relevance to liver pathophysiology and have the potential to provide significant insight into mechanisms of liver recovery and regeneration after injury.

## 1. Introduction

Drug-induced liver injury (DILI) can occur after consumption of a large variety of prescription or over-the-counter drugs as well as several supplements or natural products. It is an important public health problem, being the most common cause of acute liver failure in the Western world, predominantly due to overdose of the common analgesic acetaminophen (APAP) [1]. DILI is associated with high mortality, especially in developing countries [2], and is a common cause for withdrawal of drugs from the market rendering it very important for pharmaceutical companies involved in drug development [3]. DILI encompasses a range of hepatic pathophysiology, from idiosyncratic injury, where exact mechanisms involved in liver injury are rather nebulous [4], to liver injury caused by an APAP overdose, with relatively well-defined hepatotoxic pathways [5]. Since mechanistic details are typically scarce, idiosyncratic DILI (iDILI) is a diagnosis of exclusion and common culprits in developed countries are antimicrobials, central nervous system agents, and herbal and dietary supplements [6]. Interestingly, many of the drugs that have been implicated in iDILI can impair mitochondrial function or induce mitochondrial permeabilization, including nucleoside analog reverse transcriptase inhibitors (NRTIs) [7]. Likewise, extensive investigation of APAP-induced hepatotoxicity has established the critical role of mitochondrial dysfunction in pathophysiology [8], and this has also been demonstrated to be relevant in APAP-overdose patients [9,10]. Hence, understanding all aspects of mitochondrial biology influencing cellular responses in DILI is critical to gaining mechanistic insight into the pathophysiology of DILI.

Mitochondria have been traditionally established as the powerhouse of the cell with their role in ATP generation and bioenergetics being extensively characterized, however, it is evident that these organelles also play important roles in a variety of other cellular signaling events. Though mitochondria are typically illustrated in textbooks as being cylindrical bag-like organelles, it is now well recognized that mitochondrial morphology is dynamic. Mitochondria undergo sequences of fusion and fission, which facilitate organelle homeostasis and enable fine-tuning of mitochondrial metabolism to cellular demands. While the rate of mitochondrial dynamics, namely the frequency of fusion and fission events, varies depending on cell type, these processes occur in all cells and are necessary for maintenance of mitochondrial function. While mitochondrial dynamics have been extensively studied in neuronal cells and other cells where fewer mitochondria render them convenient to visualize by microscopy, it is recognized that these changes in mitochondrial morphology are also important in hepatocytes, which have a large number of mitochondria which could influence cellular function. This review focuses on the molecular mechanisms of mitochondrial dynamics and the relevance of this to hepatocyte survival in the context of drug-induced liver injury.

## 2. Mitochondrial Morphology

Mitochondria are double-membrane organelles with the mitochondrial matrix enclosed within the inner membrane and an intermembrane space in between the inner and outer mitochondrial membranes. The inner membrane is folded into several protrusions which project into the mitochondrial matrix, forming cristae which enlarge the surface area of the inner membrane. The mitochondrial matrix contains numerous copies of circular mitochondrial DNA, which encodes thirteen protein subunits of the respiratory complexes as well as tRNAs and rRNAs participating in mitochondrial protein synthesis [11]. The vast majority of mitochondrial metabolic enzymes are present within the mitochondrial matrix, where metabolic reactions predominate. The mitochondrial electron transport chain along with ATP synthase is localized within the inner membrane. This localization necessitates the function of the inner mitochondrial membrane as a barrier to maintain mitochondrial membrane potential for synthesis of ATP by ATP synthase using the proton gradient generated across the inner membrane by electron transport. The mitochondrial outer membrane however is relatively more permeable, though it also contains a number of transporters and molecules involved in fusion and fission.

## 3. Mitochondrial Biogenesis

Mitochondrial biogenesis is a fundamental process in cell biology which is critical for normal cellular regeneration and homeostasis. It is essential for recovery of cellular function after stress by facilitating controlled regeneration of mitochondria so that critical cellular processes such as respiration and metabolism can be maintained. Several molecular mediators controlling mitochondrial biogenesis have been identified and PGC1α is considered to be the central mediator for the process, controlling a number of downstream targets including nuclear respiratory factors (Nrfs) 1 and 2 [12,13] and transcription factor A, mitochondrial (Tfam) [14]. PGC1α, in turn is regulated by several signals of which an important one is AMP-activated protein kinase (AMPK), which acts as an energy sensor of the cell and thus works as a key regulator of mitochondrial biogenesis [14] to satisfy cellular needs. Mitochondrial biogenesis is thus a complex coordinated process which ensures that new protein synthesis in the mitochondria is coupled to that from the nucleus and functions in concert with mitochondrial fission and fusion (discussed below) to ensure proper functioning of newly synthesized mitochondria.

## 4. Mitochondrial Dynamics

Changes in mitochondrial morphology are typically restricted to fusion of two separate mitochondria (fusion) or the budding off of smaller organelles from a longer mitochondrion (fission). Coordinated cycles of fusion and fission are termed mitochondrial dynamics [15] which regulates various facets of mitochondrial function helping facilitate cellular homeostasis. Mitochondrial dynamics and mitochondrial metabolism are interrelated with both influencing each other [16,17]. Mitochondrial dynamics have been shown to be involved in metabolic regulation in cardiac and skeletal muscle cell contractions [18]. Mitochondrial morphology and metabolic state are interrelated, with increased electron chain activity being associated with elongation of the mitochondrial network in yeast [19] and energy substrates dictating mitochondrial elongation in human cancer cells [20]. Assays using isolated organelles also seem to indicate that respiratory substrates which promoted electron transport activity also led to increased mitochondrial inner membrane fusion without influencing the outer membrane [21]. Thus, conditions amenable to increased ATP production generally seem to favor elongated mitochondria [16,22], which presumably help distribution of energy throughout the cell [23]. Mitochondrial fusion has also been shown to be required for mtDNA stability in skeletal muscle and tolerance of mtDNA mutations [24] and replication of mtDNA has been linked to the proteins regulating outer and inner membrane fusion, which is necessary to maintain the stoichiometry of the protein components of the mtDNA replisome [25,26]. Mitochondrial fission on the other hand is predominantly seen in pathophysiological conditions when cells are stressed [15] and may initially be occurring as part of an adaptive mechanism linked to mitophagy (described below).

## 5. Mitochondrial Fusion Machinery

Mitochondria are dual-membrane organelles; therefore, mitochondrial fusion requires the joining of two separate membranes carried out in sequence. The outer mitochondrial membrane fusion occurs first and is followed by inner mitochondrial membrane fusion mediated by distinct GTPase proteins. Outer mitochondrial membrane fusion is mediated by the large GTPases mitofusin 1 and 2. Both proteins have an N-terminal GTPase domain, a transmembrane domain and coiled–coil heptad repeat 1 (HR1) and 2 (HR2) domains [15]. In addition, recent evidence has revealed that the C-terminal HR2 domain of human mitofusin is located within the intermembrane space (IMS) [27]. This indicates that mitofusins are inserted into the mitochondrial outer membrane through their transmembrane domains with the other N-terminal GTPase and HR1 domains projecting into the cytosol, and the C-terminal domain within the intermembrane space [27], though it has also been suggested that the protein may adopt variable topologies, with HR2 in the IMS or within the cytosol [28].

**Figure 1.**
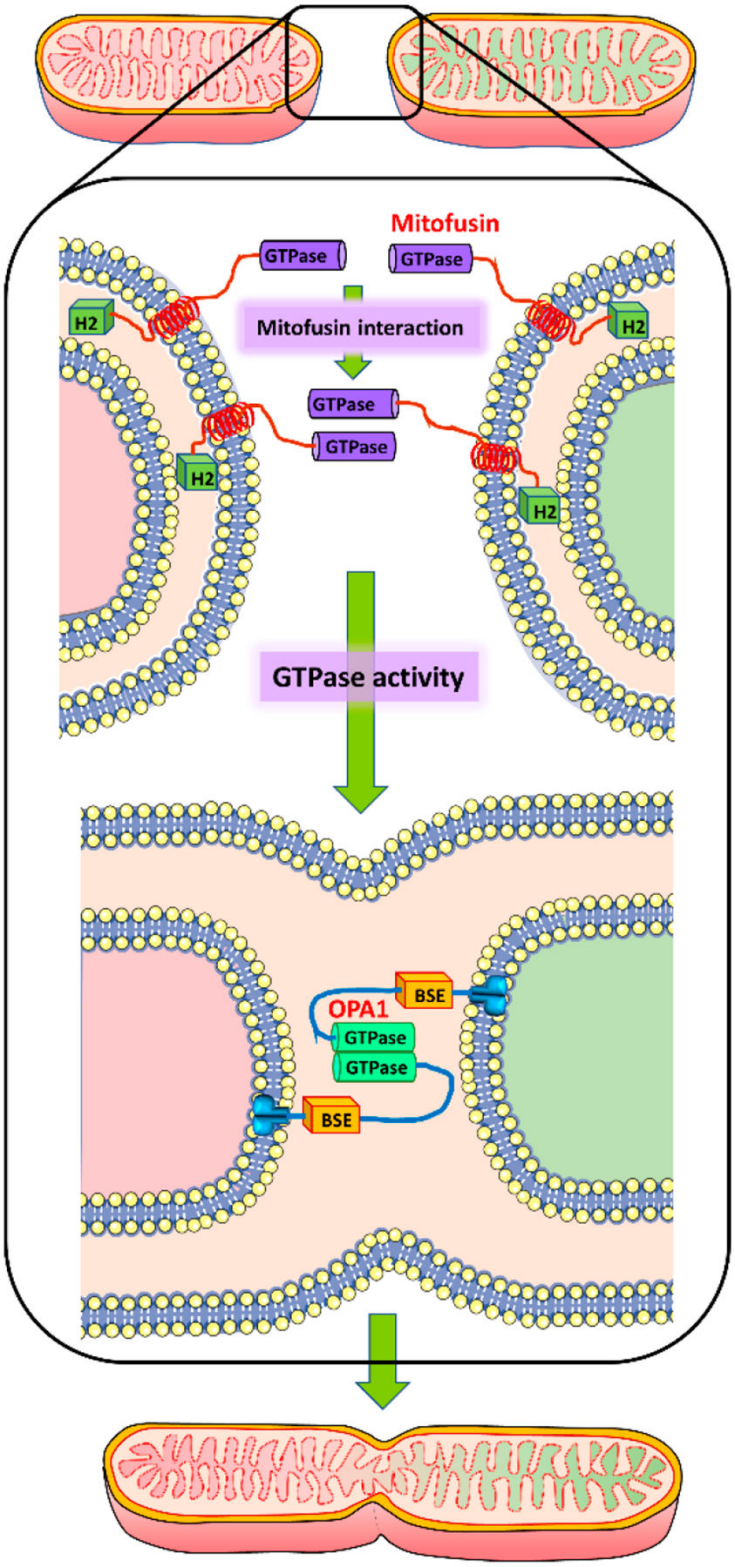
Simplified molecular mechanism of mitochondrial fusion. Mitochondrial fusion involves interaction of mitofusin molecules on the outer mitochondrial membrane of two adjacent mitochondria through their GTPase domains to facilitate outer membrane fusion through GTPase activity. Subsequent fusion of the inner mitochondrial membrane is mediated through OPA1 interaction.

Fusion of the mitochondrial outer membrane requires three distinct steps, including tethering of two organelles, docking of their outer membranes to increase the surface area of contact and decrease the distance between membranes, and ultimately fusion of the membranes due to GTPase-induced conformational change [15]. Though it was considered that initial interaction between mitofusin molecules on adjacent mitochondria occurred through their HR1 and 2 domains to initiate fusion [29], recent findings indicate that the initial interaction between two mitochondria in trans is mediated through the GTPase domains of mitofusin, and that GTP hydrolysis may drive conformational change that is required for tethering and subsequent fusion of the membranes [30]. While the exact mechanism by which GTP hydrolysis results in membrane fusion is not yet characterized, this may facilitate contact of fusogenic domains within the bilayer to facilitate the process of membrane fusion [30]. What is increasingly becoming evident, however, is the role of membrane lipids in mitochondrial fusion. It has been demonstrated that a mitochondrial phospholipase D located on the external face of mitochondria promotes trans-mitochondrial membrane adherence in a mitofusin-dependent manner by hydrolyzing cardiolipin to generate phosphatidic acid [31]. Inner membrane fusion occurs subsequent to fusion of the outer mitochondrial membrane and is mediated by another GTPase, OPA-1 in humans, which is related to the protein Mgm1 in fungi. OPA-1 is anchored to the mitochondrial inner membrane facing the inter-membrane space and is located close to the cristae [32]. Recent structural studies on Mgm1 have provided significant insight into the topology of the fusion protein, which indicate that Mgm1 consists of a GTPase (G) domain, a bundle signaling element domain, a stalk, and a paddle domain that contains a membrane-binding site [33]. The Mgm1 stalk mediates the aseembly of bent tetramers into helical filaments, which can assemble on curved membranes, where the G domains of adjacent filaments transiently dimerize and mediate a GTPase-dcpendent power stroke which can pull the filaments towards each other [33]. This facilitates scission of the inner membrane adjacent to the cristae (Figure 1), enabling complete fusion of mitochondria. Mitochondrial inner membrane lipids such as cardiolipin also facilitate this process [15].

## 6. Mitochondrial Fission

**Figure 2.**
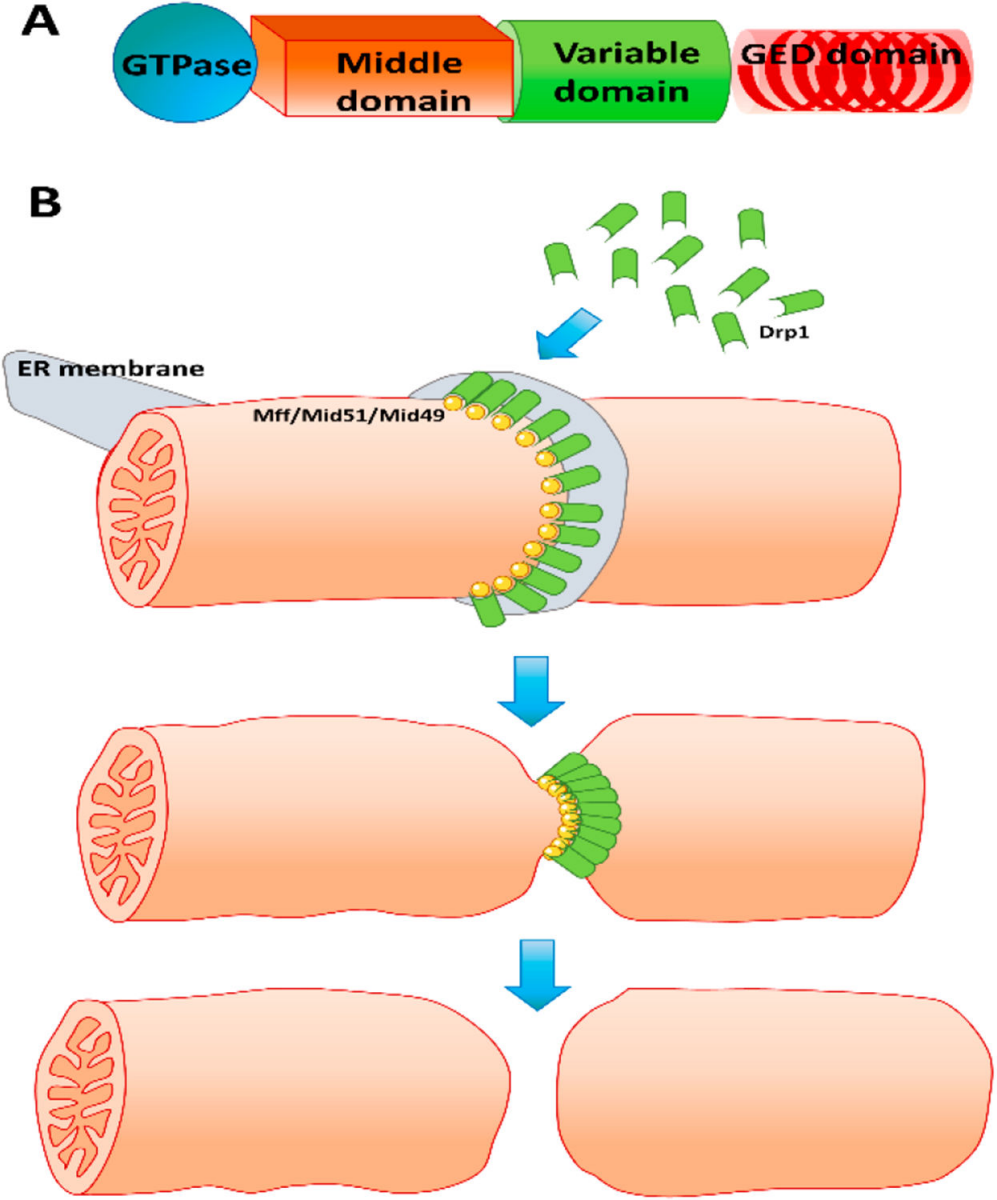
(**A**) Domain structure of the mitochondrial fission protein Drp1. (**B**) Simplified mechanism of mitochondrial fission. Mitochondrial fission is mediated by recruitment of cytosolic Drp1 onto adapter proteins such as the mitochondrial fission factor (Mff) or Mid 51/49 on the mitochondrial outer membrane at locations marked by endoplasmic reticulum membrane contact. Aggregation of Drp1 at the ER–mitochondrion contact sites facilitates GTPase-mediated constriction of the mitochondrial membrane, ultimately resulting in mitochondrial fission.

Mitochondrial fission is predominantly mediated by the large GTPase dynamin-related protein (Drp) 1, which is critical for the process [34]. Drp1 is a cytosolic protein [35] which translocates to the mitochondria and oligomerizes to form coils en the mitochondrial outer membrane to constrict mitochondria and initiate fission through GTP hydrolysis. Alternate splicing can produce up to eight different isoforms of Drp1 with varying cell-type-specific expression [36]. Drp1 has four distinct domains (Figure 2A) comprising an N-terminal GTPase domain, a middle domain, a variable domain (also called B-insert), and a C-terminal GTPase effector domain (GED) [15,36]. While the variable domain is required for binding to membranes in dynamins in general, it binds to adapter proteins on the mitochondria in the case of Drp1 [37]. The GTPase effector domain (GED) with its C-terminal coiled coil mediates multimerization of Drp1 on the membrane [38], which enables constriction (Figure 2B). Drp1 protein undergoes various post-translation modifications including phosphorylation, sumoylation, and S-nitrosylation [36], and Drp1 recruitment to the mitochondria involves binding partners such as mitochondrial fission factor (Mff) and the mitochondrial dynamics proteins 49 and 51 (MiD49 and MiD51) on the mitochondrial outer membrane [36]. The process of mitochondrial fission is a multistep process dictated initially by determination of the location on the mitochondrial outer membrane where fission is to be initiated. This seems to be influenced by a coordinated response from the endoplasmic reticulum (ER) as well as the actin cytoskeleton, where INF2-mediated actin polymerization at the ER stimulates mitochondrial calcium uptake, inner membrane constriction, and division [39]. In addition to the protein mediators discussed earlier, it is now recognized that mitochondrial membrane lipids such as cardiolipin and phosphatidic acid also play critical roles in mitochondrial fission [40,41]. In this framework, a mitochondrial phospholipase converts cardiolipin to phosphatidic acid which in concert wish saturated phospholipids can prevent Drp1-mediated fission by interfering with GTP hydrolysis [40]. In addition to coordination of mitochondrial dynamics, mitochondrial fission also plays important roles in adaptive mechanisms to cell stress such as mitophagy, where damaged or dysfunctional mitochondria are targeted for lysosomal degradation through a Parkin-mediated process [42]. Mitochondrial fission has been shown to facilitate the selective mitophagy of protein aggregates [43] and fission seems to be a necessary step for induction of mitophagy [44].

## 7. Measurement of Mitochondrial Dynamics

While general mitochondrial health and bioenergetic status can be derived through several techniques such as high content imaging of reactive oxygen species or measurement of mitochondrial respiration by instruments such as the Seahorse Analyzer, direct visual examination of changes in mitochondrial morphology typically requires microscopic techniques. Thus, mitochondrial dynamics have been predominantly evaluated in in vitro cell culture using fluorescence microscopy or high resolution confocal microscopy with mitochondrial-targeted dyes [45,46]. Additional techniques include live-cell reporter strategies to simultaneously monitor mitochondrial biogenesis and morphology using mitochondrial-targeted GFP expression under control of Nrf1 [47] as well as a 2D confocal imaging-based approach using Mitotracker^®^ staining that combines automatic mitochondrial morphology and dynamics analysis with fractal analysis in live small cell lung cancer (SCLC) cells [48]. Investigators have also used dielectrophoresis for label-free quantification of intracellular mitochondrial dynamics in human embryonic kidney cells and mouse embryonic fibroblasts by studying changes between interconnected mitochondria and those that are fragmented [49], and newer techniques using optical STED nanoscopy and an enhanced squaraine variant dye (MitoESq-635) have been used to study dynamics of mitochondrial cristae in live cells during fusion or fission at high resolution [50]. Examination of mitochondrial dynamics in vivo is rather more challenging and typically, evaluation of the expression levels of various fusion/fission proteins in tissue by QPCR or Western blotting provides an indication of the predominance of a particular mitochondrial morphology which then indicates the status of the organelle in vivo. Visualization of mitochondrial dynamics in vivo is restricted to transparent models such as the zebrafish, where transgenic approaches such as the “MitoFish” [51] or fluorescent reporters have been used [52].

## 8. Mitochondrial Dynamics in Hepatocytes

Hepatocytes play a critical role in systemic metabolism and hence are among the cells which have the highest number of mitochondria. Mitochondrial dynamics have been studied in perfused liver as well as isolated primary rat [53] and mouse hepatocytes [45]. Though discrete globular or short tubular mitochondria were evident in hepatocytes [45], few fusion events and little movement activity was noted [53], indicating that normal hepatocytes do not undergo rapid alterations in mitochondrial dynamics under physiological conditions. The caveat, however, is that these experiments were carried out in isolated primary hepatocytes under in vitro cell culture conditions where cells dedifferentiate, and mitochondrial dynamics in hepatocytes in vivo could be more active. This is all the more relevant since cells in culture are exposed to higher oxygen concentrations than hepatocytes in vivo, and oxygen tension has been shown to influence mitochondrial dynamics [54]. Chronic ethanol exposure, however, was found to effectively eliminate mitochondrial fusion and motility dynamics in primary hepatocytes [53]. As in other cell types, mitochondrial dynamics can influence metabolic functions such as gluconeogenesis in hepatocytes, which was clearly illustrated in experiments in prohibitin-deficient mice. Prohibitins are inner-mitochondrial-membrane proteins whose deficiency results in excessive proteolytic cleavage of the mitochondrial inner-membrane fusion protein OPA1 and fragmented mitochondria [55]. This was associated with lipid accumulation, abolished gluconeogenesis, and extensive liver damage [55]. However, in prohibitin-competent mice, elongation of liver mitochondria by expression of L-OPA1Δ resulted in excessive glucose production associated with increased mitochondrial respiration, demonstrating the control of mitochondrial dynamics in hepatic glucose production. The energy-modulating hormone leptin can also influence mitochondrial dynamics in hepatocytes and triggered mitochondrial fusion and alleviated high glucose-induced fatty acid accumulation in primary hepatocytes by activating mitofusin 1 [56]. The effect of bioenergetics on mitochondrial dynamics is again illustrated by the effect of calorie restriction in mice, which decreased levels of mitochondrial fission proteins such as Drp1 and Fis1 in hepatocytes without affecting fusion protein levels [57]. Mice chronically fed a high cholesterol diet showed an increase in the activation of Drp1 with decrease in OPA1, Mfn1, and Mtn2 in hepatocytes [58]. Unlike these effects on mitochondrial fusion, cellular stress typically induced mitochondrial fission in hepatocytes, which was the case in pathophysiological conditions such as nonalcoholic fatty liver disease (NAFLD), where mitochondrial fission has been suggested to play a role in etiology and transgenic inhibition of mitochondrial-fission-attenuated hepatic steatosis [59]. Preventing mitochondrial fission was also shown to protect against cholestatic liver injury, where exposure to the bile salt glycochenodeoxycholate (GCDC) rapidly fragmented mitochondria in primary mouse hepatocytes, leading to a significant increase in cell death [60]. This effect was prevented when a dominant negative fission mutant was expressed. Though GCDC is not a relevant bile acid for simulated exposure in the mouse [61], the findings are probably relevant since they held true in vivo, where a transgenic mouse model inducibly expressing a dominant-negative fission mutant specifically in the liver, showed decreased mitochondrial fission and substantially diminished ROS levels, liver injury, and fibrosis under cholestatic conditions [60]. In a mouse model of fibrosis (carbon tetrachloride), mitochondrial DNA damage resulted in dysregulation of mitochondrial fission contributing to fibrotic disposition which could be partially attenuated through inhibition of Drp1 [62]. Interestingly, endogenous expression of the mitochondrial fusion protein Mfn2 was shown to be decreased in patients with extrahepatic cholestasis, and expression of Mfn2 decreased significantly when fetal hepatocyte L02 cells were exposed to GCDC [63]. Overexpression of Mfn2 then effectively attenuated mitochondrial fragmentation and reversed the mitochondrial damage observed in GCDCA-treated L02 cells [63], further reiterating the role of perturbations in mitochondrial fusion and fission in cholestatic liver injury. Pathological conditions such as hepatic ischemia-reperfusion was also shown to increase mitochondrial fission by upregulation of Drp1, an effect attenuated by administration of the recently identified hormone irisin [64]. Mitochondrial phosphatase PGAM5-mediated mitochondrial fission was also evident in concanavalin A-induced experimental hepatitis, which was prevented by the Drp1-inhibitor Mdivi-1, which blocked mitochondrial fission, diminished hepatocyte cell death, and attenuated liver tissue damage. [65]. HBV infection was also found to shift the balance of mitochondrial dynamics toward fission and mitophagy, where HBV induced perinuclear clustering of mitochondria and induced mitochondrial translocation of Drp1 by stimulating its phosphorylation, leading to mitochondrial fission [66]. The inter-relationship between mitochondrial dynamics and metabolism is again re-iterated by the effect of mitochondrial metabolic regulators such as SIRT3, which was shown to protect against oxidative injury in hepatocytes by preventing mitochondrial translocation of Drp1 and preventing t-butyl hydroperoxide-induced mitochondrial fission [67].

## 9. Mitochondrial Dynamics and Drug- or Toxin-Induced Liver Injury

Mitochondria are central players in the etiology of a wide variety of drugs and other toxicants which produce liver injury [68]. One of the most clinically relevant drugs causing acute liver injury is acetaminophen (APAP), which, while safe at therapeutic doses, produces significant mitochondrion-mediated hepatotoxicity when consumed as an overdose [69]. This mitochondria toxicity, in part, is mediated by the phosphorylation [70] and subsequent translocation of c-Jun N-terminal kinase (JNK) to the mitochondria [71]. The role of mitochondrial dynamics in APAP-induced hepatotoxicity was initially described when elevations in Drp-1 and mitochondrial fission were identified after an APAP overdose in mice in vivo as well as in isolated mouse primary hepatocytes [45,72]. Further mechanistic investigation revealed a biphasic process whereby APAP exposure to primary mouse hepatocytes induced spheroid-shaped mitochondria, which could be reversed, before progressing towards pathological, fragmented mitochondria that was irreversible. The early change in mitochondrial morphology was caused by alterations in membrane potential, while the latter pathological change was due to decreased levels of mitochondrial fusion proteins and loss of mitochondrial phosphatidic acid [45], which can restrict DRP1-mediated fission [40]. Moreover, pharmacological inhibition of JNK phosphorylation after APAP exposure prevented the degradation of Mfn2 [45], which can be targeted by JNK for proteasomal degradation [73]. Experiments with either rat or human hepatocytes exposed to acetaminophen or diclofenac also resulted in mitochondrial fragmentation and loss of membrane potential due to decreased expression of the mitochondrial fusion proteins Mfn1 and 2, and/or Opa1. These effects were reversed by activation of AMPK, which resulted in highly fused mitochondria and an increase in ATP production [74]. Collectively, this suggests that APAP toxicity can promote mitochondrial fission directly through induction of Drp1 and indirectly by suppressing mitochondrial fusion proteins, tilting the scales towards a fragmented mitochondrial morphology. Other drugs such as rifampicin also influence Drp1 levels, where treatment of the human hepatocyte cell line QSG-7701 with the drug increased Drp1 expression and mitochondrial translocation, accompanied by cell injury [75]. Sorafenib, the targeted drug used for hepatocellular carcinoma (HCC) treatment was also found to induce mitochondrial fission, an effect amplified by IL-2 supplementation [76]. Exposure of rats to doxorubicin resulted in a decrease in primary regulators of mitochondrial fusion (OPA1, MFN1, and MFN2) with no effect on regulators of fission (DRP1 and FIS1) within the liver. This shifted the balance favoring mitochondrial fission by 24 h after exposure of doxorubicin, which likely is coupled to mitophagy and may be an adaptive response to protect against Dox-induced hepatic toxicity [77]. In addition to these drugs, several toxins have also been shown to influence mitochondrial dynamics in the liver. Triptolide, a hepatotoxic active ingredient of the Chinese herbal plant *Tripterygium wilfordii Hook F* induced mitochondrial fragmentation and change in mitochondrial dynamics in a concentration-dependent manner in human fetal hepatocyte L02 cells, by increased expression of Drp1 [78]. Inhibition of Drp1 was also found to protect against mitochondrial fragmentation induced in hepatocytes by the pyrrolizidine alkaloid senecionine, which is widely consumed as herbal medicine and food supplement [79]. In addition to these toxins, exposure to aflatoxin B1 impaired mitochondrial dynamics and increased intracellular lipid droplets in the liver of HBV-transgenic mice in vivo and the hepatitis B virus X protein (HBx)-expressing human hepatocytes, an effect mediated through upregulation of Drp1 [80].

## 10. Mitochondrial Biogenesis in Liver Injury

In addition to mediating mitochondrial dynamics and mitophagy, another component of maintenance of mitochondrial homeostasis as discussed earlier, is mitochondrial biogenesis, which typically occurs in response to cellular stress with loss of functional mitochondria and enables recovery of cellular function. Induction of mitochondrial biogenesis is mediated by upregulation of the transcription factor PGC1α [12,13], which is considered to be the master regulator for mitochondrial biogenesis. Mitochondrial biogenesis is an important factor in liver recovery and regeneration after acute injury [8,68] and it has been demonstrated that facilitation of mitochondrial biogenesis in surviving cells surrounding the necrotic area helps liver regeneration after APAP-induced liver injury [81]. Mitochondrial biogenesis is also an important mediator of chronic liver injury [82], for example, in instances of hepatitis B infection [83], and upregulation of mitochondrial biogenesis also protected against inflammatory liver injury [84]. Sepsis can also induce mitochondrial biogenesis, which was shown to be dependent on autophagy, TLR4, and TLR9 signaling in the liver [85]. Mitochondrial biogenesis has also been shown to restore oxidative metabolism in bacterial sepsis and is therefore an early and important pro-survival factor [86]. This is further illustrated by the fact that an impairment of mitochondrial biogenesis and autophagy contributed to age-dependent liver injury in experimental sepsis [87]. It was also demonstrated that the hormone irisin alleviates liver ischemia-reperfusion injury by inhibiting excessive mitochondrial fission, promoting mitochondrial biogenesis and decreasing oxidative stress [64]. A similar protective effect was seen with the lipid mediator resolvin D1, which prevented decreases in mediators of mitochondrial biogenesis such as PGC1α [88] as well as with the phosphodiesterase inhibitor cilostazol, which induced mitochondrial biogenesis by an Nrf-2- and HO-1-dependent pathway [89]. In PGC1α knockout mice treated with CCl_4_, there was enhanced liver fibrosis associated with dysfunctional mitochondrial dynamics [62]. These findings highlight that mitochondrial biogenesis and the regulation of mitochondrial fission/fusion are intimately intertwined. Additionally, impaired mitochondrial biogenesis is also implicated in metal-induced liver injury, where chronic arsenic-exposure-induced oxidative stress was shown to be mediated by decreased mitochondrial biogenesis in the rat liver [90]. Also, melatonin was shown to improve mitochondrial function by promoting MT1/SIRT1/PGC-1 alpha-dependent mitochondrial biogenesis in cadmium-induced hepatotoxicity in vitro [91]. Mitochondrial biogenesis has also been implicated in cholestatic liver injury, with transcriptional regulation of the mitochondrial biogenesis being impaired within a few hours after complete bile duct obstruction, resulting in subsequent mitochondrial dysfunction and consequent cholestatic liver injury [92]. These effects could be ameliorated by early glucocorticoid treatment, which enhanced the mitochondrial biogenesis and prevented cholestatic liver injury [93].

## 11. Mitochondrial Remodeling

Under persistent cellular stress, the alterations in mitochondrial morphology and biogenesis discussed so far can also participate in a shift of mitochondrial homeostasis to facilitate cell survival. This mitochondrial remodeling is a component of the pathophysiology in diverse diseases, including hepatic, cardiovascular, and metabolic disorders [94]. Due to the time required for adaptation, remodeling is generally associated with chronic conditions such as non-alcoholic fatty liver disease (NAFLD) and insulin resistance has been shown to be mechanistically linked to hepatic mitochondrial remodeling in this context [95]. Obesity and steatosis have been shown to promote mitochondrial remodeling that enhances respiratory capacity in the liver of ob/ob mice [96] while mitochondrial remodeling in adipose tissue was associated with obesity and treatment with rosiglitazone [97]. Mitochondrial remodeling is also a feature of chronic alcohol feeding in rats [98], and has been implicated in hepatic differentiation and dedifferentiation [99] as well as inter-organelle contact in cardiovascular pathophysiology [100]. In the context of DILI, mitochondrial remodeling could also be a feature of idiosyncratic DILI, where adaptive responses attempt to overcome the detrimental effects of the drug, and liver injury results when these are overwhelmed [101]. Though the acute nature of APAP-induced liver injury probably precludes prolonged adaptive responses, mitochondria do attempt early adaptation by altering morphology independent of canonical mitochondrial fission/fusion mechanisms in an attempt to tide over the effects of APAP on mitochondrial membrane potential [45].

## 12. Summary and Conclusions

It is now evident that aspects of mitochondrial dynamics such as fusion and fission, as well as other aspects of mitochondrial biology such as mitophagy and biogenesis, are intricately involved in maintenance of cellular homeostasis and can be significantly dysregulated in pathophysiological conditions. While it is possible that the rate of mitochondrial fusion and fission are slower in hepatocytes compared to cells from other tissues, changes in mitochondrial morphology, especially mitochondrial fission seem to be a common feature of liver injury of various etiologies. While the induction of mitochondrial fission could be an adaptive response to overcome initial stress by facilitating mitophagy, further investigations are necessary to clarify the role of this response, which could vary depending on the type of liver injury. Though a large number of the molecular mediators involved in mitochondrial dynamics have been identified and a number of mechanisms elucidated, their role in pathophysiological conditions of clinical relevance is still the focus of intense investigation. For example, though mitochondrial fission mediated by Drp1 has been identified in APAP-induced hepatocyte necrosis, its exact role in the mechanism of hepatic necrosis and its influence on other signaling pathways of programmed necrosis such as those mediated by the RIP3 kinase are still uncharacterized. Thus, the deluge of recent information on mechanisms of mitochondrial dynamics, while providing significant insight into this fascinating organelle has also opened new areas of investigation in liver pathophysiology with significant clinical relevance. In this direction, JNK reemerges as a promising therapeutic target for APAP hepatotoxicity, for its previously unrecognized role in influencing mitochondrial dynamics.
